# Intraventricular Dyssynchrony among patients with left bundle branch block

**DOI:** 10.12669/pjms.342.14103

**Published:** 2018

**Authors:** Abdul Sami, Malik Faisal Iftekhar, Imran Khan, Rafiullah Jan

**Affiliations:** 1 Dr. Abdul Sami, FCPS. Department of Cardiology, Hayatabad Medical Complex, Peshawar, Pakistan; 2 Dr. Malik Faisal Iftekhar, FCPS. Department of Cardiology, Hayatabad Medical Complex, Peshawar, Pakistan; 3 Dr. Imran Khan, FCPS. Department of Cardiology, Hayatabad Medical Complex, Peshawar, Pakistan; 4 Dr. Rafiullah Jan, FCPS. Department of Cardiology, Hayatabad Medical Complex, Peshawar, Pakistan

**Keywords:** Intraventricular dyssynchrony, Left bundle branch block

## Abstract

**Objective:**

To determine the frequency of intraventricular dyssynchrony among patients with left bundle branch block.

**Methods:**

The study was conducted at Hayatabad Medical Complex, Peshawar, from January, 2017 to July, 2017. All patients aged 18 years and above with Left Bundle Branch Block (LBBB) on ECG with or without heart failure were included in the study. Patients with valvular heart disease, predominant diastolic heart failure, acute coronary syndromes or coronary revascularization in last three months and atrial fibrillation were excluded. Tissue Doppler Imaging (TDI) parameters were measured from 2-D images in apical 4-chamber and 2-chamber views. Consecutive non-probability sampling technique was used for sample collection.

**Results:**

Our study included 159 patients. Mean age was 52 years with SD ± 2.74. Ninety-nine (62%) patients were male and 60 (38%) patients were female. One hundred and three (65%) patients had heart failure while 56 (35%) patients didn’t have heart failure. More over in our study 124 (78%) patients had Intraventricular dyssynchrony while 35(22%) patients didn’t have Intraventricular dyssynchrony.

**Conclusion:**

The incidence of Intraventricular dyssynchrony is high among patients with heart failure and left bundle branch block.

## INTRODUCTION

Left bundle branch block (LBBB) is an abnormality of cardiac conduction system which causes delayed activation of left ventricle leading to delayed left ventricular contraction compared to the right ventricle. LBBB rarely progresses to complete heart block or sudden death, and the prognosis depends on associated systemic or cardiovascular disease more than the left bundle branch block itself.^1^

Biventricular pacing / cardiac resynchronization therapy(CRT) may improve clinically significant and progressive morbidity associated with concomitant congestive heart failure.^2^ Left Ventricular (LV) dyssynchrony is an important mechanism which plays a pivotal part in progression of heart failure and ventricular remodeling. LV dyssynchrony not only affects LV systolic function but also LV diastolic function, right ventricular and left atrial function as well. In patients having complete LBBB, there is dyssynchrony between interventricular septal activation and that of LV lateral wall. The aim of CRT using biventricular pacing is to improve global LV function by synchronizing activation of the inter-ventricular septum with that of the LV lateral wall.^3^

The present study was designed to determine the frequency of intraventricular dyssynchrony in patients presenting with LBBB. LBBB is not uncommon in our population and significant morbidity and mortality in patients with LBBB are due to its progression to heart failure and failed CRT. The results of this study will give us local magnitude of the intraventricular dyssynchrony as no such study has been done in our local population presenting with LBBB. The results of this study will be very useful in generating future research strategies and for developing recommendations for routine management of patients with LBBB which may involve implantation of CRT.

## METHODS

This study was conducted at cardiology department of Hayatabad Medical Complex, Peshawar from January, 2017 to July, 2017. All patients aged 18 years and above with LBBB on ECG with or without heart failure were included in the study. Patients with valvular heart disease, predominant diastolic heart failure, acute coronary syndromes or coronary revascularization in last three months and atrial fibrillation were excluded from the study.

All patients were subjected to detailed history and clinical examination. All patients were subjected to tissue Doppler imaging and two-dimensional Echocardiography. Tissue Doppler imaging (TDI) was performed by using apical four chambers, apical two chamber and parasternal long axis views for the long axis motion of the ventricles. Two-dimensional echocardiography with TDI- color imaging views was optimized for pulse repetition frequency, color saturation, and sector size and depth and to allow highest possible frame rate. Pulsed-wave TDI velocities of long-axis wall motion was assessed in apical views during end-expiratory apnea, with sample volume of 5 mm positioned in the center of the analyzed segment. Intraventricular dyssynchrony was determined as the difference between the longest and shortest electromechanical coupling times in the opposing basal septal, lateral, anterior, inferior and posterior segments of the left ventricle. Any difference of 40msec or more was considered indicative of presence of intraventricular dyssynchrony (on Tissue Doppler Imaging). All these TDI and ECHO procedures were done by single experienced cardiologist having minimum of seven years of experience.

All the above-mentioned information including name, age, gender and address was recorded in a pre-designed proforma. Strict exclusion criteria had been followed to control confounders and bias in the study results.

Data was analyzed using SPSS version 20. Quantitative variable like age was described in terms of means ± standard deviation. Categorical data like gender, heart failure and Intraventricular dyssynchrony was described in the terms of frequency and percentages. Intraventricular dyssynchrony was stratified among age, gender and heart failure to see the effect modifications. Chi square was used and a value less than 0.05 were considered significant.

## RESULTS

Among 159 patients evaluated 99 were male with mean age 52 ±2.7 standard deviation (SD). Dyssynchrony distribution among patients with LBBB showed interventricular dyssynchrony in 124 patients (78%) while non interventricular dyssynchrony accounted for 35 (22%). Baseline characteristics including age, gender, heart failure was statistically significant among both groups (Table-I). Stratification of patients characteristics based on intraventricular dyssynchrony is shown in Table-I.

**Table-I:** Summary of Patient Characteristics (n=159).

	IVD present	IVD Not present	p-value
***Age***			
30-40 years	19	0	0.0251
41-50 years	40	11	
51-60 years	44	12	
61-70 years	21	12	
***Gender***			
Male	77	22	0.0139
Female	47	13	
***Heart Failure***			
Present	80	44	0.0279
Not Present	23	12	

## DISCUSSION

Left bundle branch block (LBBB) is an abnormality of cardiac conduction system which causes delayed activation of left ventricle leading to delayed left ventricular contraction compared to the right ventricle.^1^ LBBB in children is associated with cardiovascular disease or surgery and is not observed in the general population. Left bundle branch block may occur in as many as 20% of individuals after aortic valve replacement.^3^ Progression to complete heart block or sudden death is rare, and the prognosis depends on associated systemic or cardiovascular disease more than the left bundle branch block itself. Biventricular pacing may improve clinically significant and progressive morbidity associated with concomitant congestive heart failure.^4^

Our study showed the incidence of Intraventricular dyssynchrony in LBBB was 124(78%).

Similar results were found in another study conducted by Rao HB et al.^5^, 72% of patients with LBBB demonstrated intraventricular dyssynchrony. Overall 37% had lateral wall delay and 16% had septal delay.^5^

In another study conducted by Malhotra S et al.^6^, the mean Left Ventricular Ejection Fraction (LVEF) of the cohort was 27±6% and LBBB was present in 33 patients (22%). LV dyssynchrony was present in 123 patients (90%). There were no differences in prevalence of LV dyssynchrony (91% vs. 90%, p=0.86) or its severity (Pulse Subtraction Doppler (PSD): 45±19 vs. 40±18, p=0.17; Histogram Bandwidth (HBW): 138±69 vs. 132±72, p=0.67) among those with and without LBBB. Infarct size, as assessed by the summed rest score (SRS), LVEF and female gender were significant predictors of LV dyssynchrony in a multivariable logistic regression analysis. LBBB and QRS duration did not predict LVD. Another study conducted by Ignasi A et al.^7^, 70% of patients with LBBB demonstrated intraventricular dyssynchrony with 41% having lateral wall delay and 20% having septal delay.

## CONCLUSION

Our study concludes that in our setup the incidence of Intraventricular dyssynchrony was high among patients presenting with left bundle branch block. LV dyssynchrony is not the only mechanism responsible for less efficient LV pump but there are other mechanisms in play as well. Further studies are needed to fully understand the mechanism of LV pump failure and develop strategies to ameliorate heart failure by modifying factors responsible.

### Author`s Contribution

**AS** conceived, designed and did statistical analysis and manuscript writing.

**MFI, IK and RJ** did data collection and editing of manuscript.
